# A New Year at *PLoS Medicine*: Maintaining a Focus on the World's Health Priorities and Identifying the Gaps

**DOI:** 10.1371/journal.pmed.1001168

**Published:** 2012-01-31

**Authors:** The PLoS Medicine Editors

## Abstract

In the January editorial, the *PLoS Medicine* editors review the journal's contents in light of its mission and scope, and call for papers on specific topics.

Almost three years ago [1], *PLoS Medicine* announced that the journal would both reflect and draw attention to the world's health priorities. In so doing, our intention was to give the highest priority to studies and commentary that advanced understanding of the conditions and risk factors that contribute most substantially to loss of health and life on a global scale. This approach to redefining the journal's mission has allowed *PLoS Medicine* to capitalize on one of our key underlying values: open access. By prioritizing publication of studies that have far-reaching relevance in global health, and enabling access to those findings by all, *PLoS Medicine* hopes to address health inequities specifically. We also continued to publish on important cross-cutting topics in clinical practice, health policy, and the conduct of research, especially taking a lead in the ethics of medical publishing as demonstrated by cases from the pharmaceutical and tobacco industries.

So, what impact has this change in our scope had? As noted in a previous editorial [2] it did not take long for the journal's content to come to reflect its redefined mission. To that end, we have found that difficult judgments are sometimes required on the quality of a given field at a given time, in order to ensure that questions that lack a mature body of research are not disadvantaged. As a result, over the past three years we have published extensively on topics and issues that may not be traditionally seen as the prerogative of medical journals. For example, in the past year our most highly accessed research articles have included work analyzing the factors associated with violent deaths among Iraqi civilians [3]; a report of medical records revealing evidence of doctors' complicity with torture of individuals held at Guantánamo Bay [4]; a study looking at the likely effect of scaling up diarrhea prevention efforts [5]; and an investigation revealing the extent of off-label marketing by pharmaceutical companies [6]. The *PLoS Medicine* magazine section has championed important but neglected causes and themes in global health, in particular through our collections on migration and health [7], water and sanitation [8], health systems research, global health estimates [9], and ghostwriting [10].

However, a review of the work submitted to and published by *PLoS Medicine* has identified important gaps in major research questions on the global burden of disease. The lack of available research on some conditions will have multiple causes; it may reflect inequities in the distribution of research funding [11] the lack of a robust research infrastructure in many parts of the world; and, of course, the decisions of researchers as to whether and where to publish. However, we recognize that by actively choosing to publish on particular topics, journals can draw attention to under-researched questions and help ensure that neglected issues are elevated on the agenda of political and scientific discussions. Therefore, we have identified as priority areas for 2012 the following topics, for which we would like to see more submissions of high-quality clinically and policy-relevant research:

- Respiratory conditions (including chronic obstructive pulmonary disease)
- Stomach, colorectal, liver, and lung cancers
- Vision and hearing disorders
- Injuries

Additionally, because they contribute to a substantial burden of ill health worldwide, we are interested in papers on:

- Unmet contraception needs
- Unsafe sex
- Childhood sexual abuse
- Illicit drug use

And finally, as before, we want to continue to encourage the submission of research addressing more cross-cutting issues of broad importance in global health and health policy, for example in relation to:

- Human rights
- Quality of care
- Methodological questions
- Outputs of research prioritization initiatives

When we set out to take an evidence-based approach to our scope we never intended to be restricted to a single list of conditions but rather to be guided by consideration of what “really matters” in global health, at a population level. We recognize that the world changes fast. New estimates soon to be released from the Global Burden of Disease study will provide an updated picture of health risks and conditions and give insight into whether previous predictions of health transitions [12] are accurate. We intend to be informed by the evolving pattern of health transitions around the globe.

We feel that we have succeeded in our aims of three years ago: to highlight neglected issues, to promote globally relevant questions in health, to scrutinize ethics in biomedical publishing, and to advocate for integrity in research. However, it is not enough to simply raise awareness among our readership by publishing on issues that really matter in global health; it is now essential to leverage this awareness to bring about change in health care priorities globally.
